# Editorial: Involvement of dendritic cells in gastrointestinal cancer

**DOI:** 10.3389/fimmu.2023.1178075

**Published:** 2023-03-17

**Authors:** Ling Ni, Jingtao Chen, Mi Deng, Haidong Tang, Musheng Bao

**Affiliations:** ^1^ Institute for Immunology and School of Medicine, Tsinghua University, Medical Research Building, Beijing, China; ^2^ Laboratory for Tumor Immunology, the First Hospital of Jilin University, Changchun, China; ^3^ Peking University International Cancer Institute, Peking University, Beijing, China; ^4^ School of Pharmaceutical Sciences, Tsinghua University, Beijing, China; ^5^ Biology Group, Nona Biosciences, Natick, MA, United States

**Keywords:** dendritic cells, gastrointestinal cancer, DC differentiation, cancer immunotherapy, targeting DCs

Immunotherapy has revolutionized cancer treatment over the past decade with remarkable results in terms of durable remission and extended survival in some patients. However, the response rate to current immunotherapy in patients with gastrointestinal (GI) cancer remains relatively low. For successful anti-cancer immune responses, the cancer-immunity cycle needs to be initiated to activate neoantigen-specific T cells and eliminate tumor cells. Dendritic cells (DCs) play a crucial role by processing neoantigens and presenting them to T cells. Therefore, targeting DCs has a significant potential for cancer immunotherapy. The current collection, “*Involvement of Dendritic Cells in Gastrointestinal Cancer*,” covers recent studies on the involvement of DCs in GI cancer, including topics such as the differentiation of thymic DCs, immunological tolerance induced by DCs in tumors, the role of DCs in GI cancer, DC-based cancer immunotherapy, and strategies for targeting DCs in immunotherapy.

Tumor progression blocks the transition of double negative (DN) early T-cell progenitors in T cell maturation, instead leading to DN-T-cell differentiation into DCs (Guha et al.). Mechanistically, thymically-expressed IL-10 promotes the interaction between thymic stromal cells and Notch1(low) DN2-T cells, thus facilitating these DN2-T cells to differentiate toward thymic DCs, which thus limits the protective adaptive immune repertoire. The development of plasmacytoid DCs and their roles in a variety of malignancies, including GI cancers, have been summarized by Zhou et al.

Inhibitory immune checkpoint molecules on DCs have been shown to be involved in diminishing the efficacy of DC-mediated anti-tumor immune responses. Ghorbaninezhad et al. showed that silencing CTLA-4 using siRNA induces DC maturation, which leads to increased T cell proliferation and cytokine production. On the other hand, cancer-associated fibroblasts (CAFs) actively participate in tumor development and affect treatment responses. Berzaghi et al. showed that CAFs induce a tolerogenic phenotype in DCs, promoting the downregulation of the DC signature, activation markers, and functions. Furthermore, certain radiation regimens can reverse the CAF-mediated immunosuppressive effects.

Subtil et al. established an organotypic 3-dimentions co-culture system to recapitulate and untangle interactions between DCs and patient-derived CRC organoids. They demonstrated high viability and extensive interactions between DCs and tumor organoids, which control the expression of activation markers on DCs and their ability to activate T-cells. In another study, by analyzing the data from a colon adenocarcinoma (COAD) cohort receiving immunotherapy, Zhou et al. found that the IL-1 signaling mutated-type group has higher infiltration levels of activated DCs, M1 macrophages, neutrophils, activated natural killer cells, activated CD4^+^ memory T cells, and CD8^+^ T cells than IL-1 signaling wild-type groups. These findings suggest that IL-1 mutation may be an independent biomarker for prognosis in patients with COAD receiving immunotherapy.

Advances in DC-based immunotherapy and clinical trials that indicate therapeutic efficacy and toxicity related to each vaccine have been reviewed by Ni et al. At the single-cell level, Wang et al. summarized the classification and development trajectory of DCs in GI cancer with a focus on the interaction of DCs with T cells and their effects on immunotherapy response. Newly identified tumor-infiltrating DCs and their potential functions in anti-tumor immunity have also been summarized. In order to offer more detailed evidence and novel opinions to enhance the development of a personalized neoantigen-based DC vaccines for pancereatic cancers (PCs), Zhang et al. summarized the advance of the neoantigen, neoantigen-based vaccines, and DC-based vaccine with the emphasis of the combination of the neoantigen and DC-based vaccine.

Rb9 is a cyclic VHCDR3-derived peptide from the RebMab200 antibody targeting a NaPi2B phosphate-transport protein. Rb9 showed anti-tumor activity in syngeneic mice, which was mainly due to increased CD8^+^ T infiltration and decreased intratumoral Foxp3^+^ T cells (Machado et al.). Human DCs showed increased expression of activation markers after exposure to Rb9.

Taken together, studies on DCs in GI cancer have achieved encouraging results. Nonetheless, a better understanding of the phenotypes and functions of DCs is required before we can properly target DCs and improve the overall response rate in GI cancer immunotherapy.

## Author contributions

All authors listed have made a substantial, direct, and intellectual contribution to the work and approved it for publication.

